# Major Shift of Toxigenic *V*. *cholerae* O1 from Ogawa to Inaba Serotype Isolated from Clinical and Environmental Samples in Haiti

**DOI:** 10.1371/journal.pntd.0005045

**Published:** 2016-10-07

**Authors:** Meer T. Alam, Shrestha S. Ray, Camille N. Chun, Zahara G. Chowdhury, Mohammed H. Rashid, Valery E. Madsen Beau De Rochars, Afsar Ali

**Affiliations:** 1 Emerging Pathogens Institute, University of Florida, Gainesville, Florida, United States of America; 2 Department of Environmental and Global Health, College of Public Health and Health Professions, University of Florida, Gainesville, Florida, United States of America; 3 Department of Microbiology and Cell Science, College of Agricultural and Life Sciences, University of Florida, Gainesville, Florida, United States of America; 4 Department of Health Services Research, Management, and Policy, College of Public Health and Health Professions, University of Florida, Gainesville, Florida, United States of America; University of California San Diego School of Medicine, UNITED STATES

## Abstract

In October of 2010, an outbreak of cholera was confirmed in Haiti for the first time in more than a century. A single clone of toxigenic *Vibrio cholerae* O1 biotype El Tor serotype Ogawa strain was implicated as the cause. Five years after the onset of cholera, in October, 2015, we have discovered a major switch (ranging from 7 to 100%) from Ogawa serotype to Inaba serotype. Furthermore, using *wbeT* gene sequencing and comparative sequence analysis, we now demonstrate that, among 2013 and 2015 Inaba isolates, the *wbeT* gene, responsible for switching Ogawa to Inaba serotype, sustained a unique nucleotide mutation not found in isolates obtained from Haiti in 2012. Moreover, we show that, environmental Inaba isolates collected in 2015 have the identical mutations found in the 2015 clinical isolates. Our data indicate that toxigenic *V*. *cholerae* O1 serotype Ogawa can rapidly change its serotype to Inaba, and has the potential to cause disease in individuals who have acquired immunity against Ogawa serotype. Our findings highlight the importance of monitoring of toxigenic *V*. *cholerae* O1 and cholera in countries with established endemic disease.

## Author Summary

For the first time in 100 years, in October 2010, cholera caused by toxigenic strains of *V*. *cholerae* was introduced in Haiti. Conventional and genetic analysis revealed that a single clone of *V*. *cholerae* O1 biotype El Tor, serotype Ogawa strain was brought to Haiti by Nepalese Peace-Keeping troops. These troops arrived in January of that year to provide humanitarian assistance following Haiti’s deadliest earthquake. Subsequently our team has monitored the cholera epidemic by acquiring clinical and environmental samples to assess whether or not the pathogen was able to establish its environmental reservoirs within the country, and whether *V*. *cholerae* could undergo evolutionary changes in order to adapt to the stressors imparted by human gastrointestinal tract and environmental aquatic reservoirs. In this study we show that over the past 5 years the initially-introduced and circulating *V*. *cholerae* Ogawa serotype has significantly shifted to Inaba serotype by sustaining multiple mutations in *wbeT* gene, the gene known to mediate serotype shift. Our findings suggest that the new Inaba serotype may be replacing the Ogawa serotype in order to evade Ogawa-induced host immunity and thereby causing cholera potentially among individuals who may previously have suffered from cholera. Our study highlights the importance of ongoing monitoring of cholera in Haiti.

## Introduction

After being absent for 100 years, cholera was introduced into Haiti in October, 2010 by Nepalese Peace-Keeping troops providing assistance to Haiti following the 2010 earthquake [1]. The initial cholera epidemic was caused by a single clone of toxigenic *V*. *cholerae* O1 biotype El Tor, serotype Ogawa [1, 2]. Although the incidence of cholera in Haiti has significantly decreased following the initial 2010–2011 epidemic wave, case numbers are again increasing with 22,511 cholera cases being reported in Haiti through epidemiological week 38 (beginning of October) of 2015 (http://www.paho.org/hq/index.php?option=com_docman&task=doc_view&Itemid=270&gid=31956&lang=en). Our group has been conducting cholera surveillance in Haiti at the field level beginning three weeks after the onset of the initial outbreak [2]. Our surveillance has included clinical cholera (de-identified) and sentinel aquatic environmental surveys primarily in the Gressier (rural) region of Haiti beginning in May, 2012 [3]. Our goals have been to assess the long-term impact of cholera in Haiti, particularly to determine if the microorganism is able to establish aquatic reservoirs, and to evolve over time in response to patients’ acquired immunity to the pathogen, and to environmental stressor(s). As part of that surveillance, we have isolated a large number of toxigenic *V*. *cholerae* strains from stool samples of suspected cholera patients admitted to cholera treatment centers (CTCs) located in Gressier, Jacmel and Petti Goave regions; and collected monthly fresh water samples from 15 sites to identify toxigenic *V*. *cholerae* O1 strains [3, 4]. Using whole genome sequencing and SNP analysis we have compared a large number of clinical isolates collected in 2010, 2011 (obtained from Gen Bank accession number) and 2012, and found that 2011 and 2012 strains have measurably evolved from the original clonal strains isolated immediately after 2010 cholera outbreaks [5]. Our observations suggest that toxigenic *V*. *cholerae* O1 strains have established environmental reservoirs in Haiti, and that more recent strains have measurably evolved from initial clone demonstrating potential adaption to the immune response of Haitians exposed to the initial cholera pathogen.

As noted above *V*. *cholerae* O1 biotype El Tor serotype Ogawa was responsible for initial cholera outbreak in Haiti; however, *V*. *cholerae* serogroup O1 has three serotypes, Ogawa, Inaba and Hikojima and previous studies suggest that the microorganism can undergo unequal and reciprocal interconversion among these serotypes in response to stressors [6]. *V*. *cholerae* has two life cycles, surviving in aquatic reservoirs and in human gut where it causes cholera, and the organism faces selective pressures in both life stages [7, 8]. These selective pressures can drive (a) the evolution to a completely new serogroup of *V*. *cholerae* as evidenced by the emergence of *V*. *cholerae* O139 serogroup in 1992 [9, 10] and (b) can also result in unequal reciprocal interconversion among the three serotypes (Ogawa, Inaba and Hikojima) [6]. Evidence from *in vitro*, *in vivo* studies, as well as anecdotal evidence suggest that *V*. *cholerae* can switch/interconvert from Ogawa to Inaba or Inaba to Ogawa serotype, and rarely to Hikojima in response to selective pressure(s) [6, 11, 12].

The *wbeT* gene (formerly known as *rfbT* gene), represents a gene cluster that encodes for a transferase responsible for the expression of the B determinant of O- antigen that is required for the expression of Ogawa-specific serotype [6, 11]. Nonsynonymous mutations, including point mutations, insertions, and deletions (INDELs) in *wbeT* gene result in the expression of a modified or truncated protein that forms the C determinant of O-antigen found in the Inaba-specific serotype. The expression of both the B and C determinants results in the expression of Hikojima serotype that is very unstable and more prone to reciprocal serotype interconversion [6, 11]. In mice treated with anti-Ogawa serum, and then challenged with Ogawa strains select for an Inaba serotype suggesting that anti-Ogawa antibody promoted the emergence of the Inaba serotype [12]. Switching from Inaba to Ogawa serotype has also been reported, but is rare [13].

Several recent studies have suggested that *V*. *cholerae* O1 Ogawa serotype introduced in Haiti in 2010 switched (at a very low frequency; ~1.5% of total *V*. *cholerae* isolates) to Inaba serotype [14–16]. Using whole genome sequence analysis, Katz et al. [16] demonstrated that all five of their *V*. *cholerae* serotype Inaba strains isolated in Haiti in 2012 sustained a G→A substitution (at position 493) in *wbeT* gene leading to a premature stop codon at position 493 resulting in a truncated protein, a hallmark of *V*. *cholerae* Inaba serotype [6].

In contrast to these previous reports, we have found very high-frequency switching of *V*. *cholerae* O1 from Ogawa to Inaba serotype among isolates collected from stool and water samples beginning in October 2015 in Haiti. We also report that our Inaba serotype isolates have both substitution and deletion mutations in the *wbeT* gene at different positions than those described in earlier report [16].

## Materials and Methods

For isolation of toxigenic *V*. *cholerae* strains, de-identified stool samples were collected from the patients admitted to diverse cholera treatment facilities (hospitals and cholera treatment centers [CTC]) and the majority of samples were transported within 2 hours of collection to the UF field laboratory housed in the Gressier region of Haiti. If transportation was not possible within 2 hours, stool samples were stored in the Carry-Blair (CB) transport medium [17]. Environmental samples (water) were collected monthly from the 15 fixed sites and the samples were processed for the identification of *V*. *cholerae* as described previously [3]. Briefly, both stool and water samples were inoculated in alkaline peptone water (APW) and the cultures were incubated at 37 and 40°C at different incubation time. Subsequently, a loopful of culture was streaked onto TCBS agar for obtaining isolated colonies. Eight to ten yellow colonies with diverse morphologies grown on each TCBS agar were transferred to L-agar and the plates were incubated overnight at 37°C. Cultures grown on L-agar was tested for oxidase activity and oxidase-positive colonies were tested for toxigenic *V*. *cholerae* O1 or O139 using O1-specific polyvalent and O139-specific antisera (DENKA SEIKEN USA Inc., Campbell, CA), respectively. Polyvalent serum positive *V*. *cholerae* O1 isolates were further serotyped using antiserum specific to Ogawa and Inaba. Seropositive *V*. *cholerae* was then subjected to colony PCR to determine the presence of *ompW* and *toxR* genes specific for *V*. *cholerae* species as described previously [2].

To amplify and sequence *wbeT* gene of *V*. *cholerae* isolated from clinical and environmental samples in Haiti, chromosomal DNA of each isolate of interest was extracted using GenElute Bacterial Genomic DNA kits (Sigma-Aldrich, St. Louise, MO) following manufacturer’s recommendation. Two convergent PCR primers, including WF1 (5’-GATGTTCATGCGGTTTCCGT-3’) and WR1 (5’-CAGGAATTCACAGCACATCGC-3’) were used to amplify the entire *wbeT* gene. Briefly, PCR was performed using a100 μl mixture containing 10 μl of 10X Buffer, 6 μl of 50 mM MgCl_2_ (Invitrogen, Carlsbad, CA)_,_ 1 μl of 10 mM deoxynucleotide triphosphates (New England BioLabs, Ipswich, MA), 1 μl of 5 U Taq DNA polymerase (Invitrogen, Carlsbad, CA) and 20 μm of each primer. Initial denaturation was accomplished by heating to 95°C for 5 min; followed by 30 cycles of 95°C for 1 min, 60°C for 1 min to anneal the primers, 72°C for 1 min to extend the annealed primers, and 72°C for 5 mins for final extension. 10 μl of PCR products were then electrophoresed on 1% agarose gel to visualize the band specific for *wbeT* gene. The remaining PCR product (90 μl) was purified using QIAquick PCR purification kit (Qiagen, Valencia, CA) and the purified product was sequenced at Interdisciplinary Center for Biotechnology Research (ICBR) in the University of Florida. Mutation analysis and sequence comparison were performed using the multiple sequence alignment tool MEGA 7 (Molecular Evolutionary Genetic Analysis) [18]. All the *wbeT* sequences obtained from this study were deposited in GenBank (accession no. KX379810-KX379851).

### Ethics statement

Collection of stool samples was routine. Patients did not provide informed consent, as collections and culturing of samples was part of routine medical care. Stool samples potentially containing *V*. *cholerae* strains, with all personal identifiers removed, were provided to the study investigators for further analysis. This study protocol was reviewed by the University of Florida IRB, and approved as a non-human study involving de-identified samples.

## Results and Discussion

### High-frequency switching of *V*. *cholerae* Ogawa to Inaba serotype in Haiti

**Fig 1:**
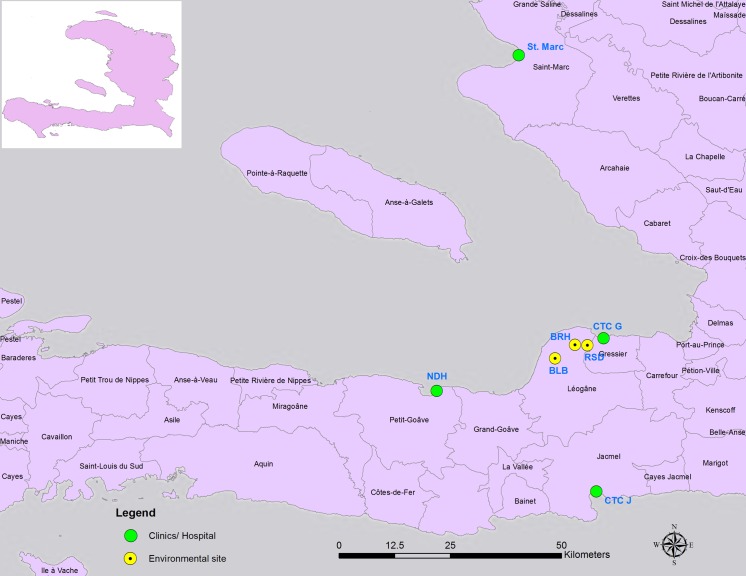
Map displaying clinical and environmental sites wherein we obtained toxigenic *V*. *cholerae* positive isolates. Green circular shapes reflect clinical cholera sample collection sites while yellow circular shapes point to sentinel environmental sites yielding toxigenic *V*. *cholerae* O1 strains. NDH, Notredame Hospital de Petit-goave; CTC J, Cholera Treatment Center in Jacmel; BLB, Bay Larion Bridge; BRH, Brach; RSD, Reserved; CTC G, Cholera Treatment Center in Gressier; St. Marc, St Marc Hospital.

Several earlier reports demonstrated a very low level (< 1.5%) of isolation of *V*. *cholerae* O1 Inaba serotype between 2011 and 2012 in Haiti [15, 16, 19]. In our clinical survey that began in May, 2012, we isolated several hundred *V*. *cholerae* O1 Ogawa serotype collected from different cholera treatment centers and clinics in Haiti. Consistent with other reports [16, 19], we isolated two *V*. *cholera*e O1 serotype Inaba strains from two distinct sites, including Notredame Hospital (NDH) in Petit Goave and cholera treatment center (CTC) in Jacmel, Haiti (Fig 1) in the months of July and November, 2013, respectively. However, we strikingly observed a major switching (7–100%) from Ogawa to Inaba serotype beginning in October, 2015 (Table 1), exactly 5 years after the onset of cholera in Haiti in October, 2010. We observed this shift of Ogawa to Inaba serotype in isolates collected from two different sites that are 37.7 miles (Fig 1) apart (from Petit Goave to Jacmel) suggesting that this phenomenon of serotype conversion is widespread in Haiti.

**Table 1:** Comparative distribution of toxigenic *V*. *cholerae* O1 serotypes Ogawa and Inaba isolated from cholera treatment centers in Haiti.

Sampling month	Sampling sites	*V*. *cholerae* O1 strains	Ogawa serotype (%)	Inaba serotype (%)
July, 2015	CTC Jacmel, Haiti	1	1 (100)	0 (0)
	CTC Gressier, Haiti	0	0 (0)	0 (0)
August, 2015	CTC Jacmel, Haiti	1	1 (100)	0 (0)
	CTC Gressier, Haiti	1	1 (100)	0 (0)
September, 2015	CTC Jacmel, Haiti	8	8 (100)	0 (0)
	CTC Gressier, Haiti	0	0 (0)	0 (0)
October, 2015	CTC Jacmel, Haiti	28	26 (92.86)	2 (7.14)
	CTC Gressier, Haiti	5	3 (60)	2 (40)
November, 2015	CTC Jacmel, Haiti	33	16 (51.5)	15 (48.5)
	CTC Gressier, Haiti	25	0 (0)	25 (100)
December, 2015	CTC Jacmel, Haiti	5	3 (60)	2 (40)
	CTC Gressier, Haiti	4	2 (50)	2 (50)

CTC, cholera treatment center.

Although we do not have definitive evidence, we provide two potential underlying explanations for Ogawa to Inaba switching: first, during this five years of ongoing cholera in Haiti, individuals of that country may have acquired adequate anti-Ogawa antibody in their sera to protect themselves from cholera caused by *V*. *cholerae* Ogawa serotype. The elevated level of anti-Ogawa antibody could result in an adaptive switch of *V*. *cholerae* from Ogawa to Inaba serotype enabling this pathogen to continue to propagate in the human host and maintain its continuity in Haiti. Second we are also finding evidence of the shift form Ogawa to Inaba in environmental samples: while this may reflect contamination of environmental sources by isolates from humans, there is also the possibility that the driver for the shift in serotype arose from environmental stressors, and in turn was communicated to humans. Continued analysis of both clinical and environmental strains will be necessary to gain a better understanding of the selection factors underlying this shift.

### Genetic analysis of *wbeT* gene

Mutation(s) in *wbeT* gene occurring in O-antigen biosynthetic genetic component of *V*. *cholerae* O1 has been implicated in the interconversions among Ogawa, Inaba and Hikojima serotypes [6, 11]. To determine whether original clone of *V*. *cholerae* O1 serotype Ogawa had undergone any changes in *wbeT* gene across time and space following the microorganism’s introduction into Haiti, we have compared the *wbeT* gene sequences of representative isolates of *V*. *cholerae* O1 Ogawa strains, collected from both clinical and environmental sources, to that of a prototype Ogawa serotype strain, NIH41 [20]. The Ogawa isolates included in this comparison, included strains of: 2010 (n = 2 clinical); 2012 (n = 4: 2 clinical, and 2 environmental isolates); 2013 (n = 4: 2 clinical, and 2 environmental isolates); 2014 (n = 4: 2 clinical, and 2 environmental isolates); and 2015 (n = 2 clinical) (Table 2 and S1 Table). Compared to NIH41 *wbeT* sequence, Haitian isolates replaced the A for C in position 553 in that gene resulting in a silent substitution of lysine to glutamine as reported previously [13]. We did not observe any other changes in *wbeT* gene of *V*. *cholerae* O1 Ogawa isolates across space and time (Table 2).

**Table 2:** Analysis of mutations occurring in *wbeT* gene in *V*. *cholerae* O1 strains isolated from clinical and environmental samples in Haiti.

Serotype	Total no. of strains	Isolation time	Sample source	Distinct mutation(s) occurred relative to NIH41^a^
Ogawa	2	November, 2010	C	A→ C at 553 position changes lysine to glutamine.
	2	June, 2012	C	A→ C at 553 position changes lysine to glutamine.
	1	May, 2012	E	A→ C at 553 position changes lysine to glutamine.
	1	June, 2012	E	A→ C at 553 position changes lysine to glutamine.
	1	August, 2013	C	A→ C at 553 position changes lysine to glutamine.
	1	December, 2013	C	A→ C at 553 position changes lysine to glutamine.
	1	June, 2013	E	A→ C at 553 position changes lysine to glutamine.
	1	October, 2013	E	A→ C at 553 position changes lysine to glutamine.
	1	June, 2014	C	A→ C at 553 position changes lysine to glutamine.
	1	October, 2014	C	A→ C at 553 position changes lysine to glutamine.
	1	May, 2014	E	A→ C at 553 position changes lysine to glutamine.
	1	June, 2014	E	A→ C at 553 position changes lysine to glutamine.
	2	November, 2015	C	A→ C at 553 position changes lysine to glutamine.
Inaba	1^b^	March, 2012	C	A→ C at 553 position changes lysine to glutamine and G→ T at 493 position leads to stop codon at 493.
	1	July, 2013	C	A→ C at 553 position changes lysine to glutamine and A→ G at 395 position change glycine to glutamate.
	1	November, 2013	C	A→ C at 553 position changes lysine to glutamine and deletion of C at 474 leads to stop codon at 481.
	4	October, 2015	C	A→ C at 553 position changes lysine to glutamine and deletion of C at 329 position leads to stop codon at 385 position.
	2	November, 2015	E	A→ C at 553 position changes lysine to glutamine and deletion of C at 329 position leads to stop codon at 385 position.
	21	November, 2015	C	A→ C at 553 position changes lysine to glutamine and deletion of C at 329 position leads to stop codon at 385 position.
	3	December, 2015	C	A→ C at 553 position changes lysine to glutamine and deletion of C at 329 position leads to stop codon at 385 position.

C, clinical (stool) samples; E, environmental (water) samples.

^a^*wbeT* gene sequence was retrieved from GenBank accession no. X58834 and reference no. [11] for genetic comparative analysis.

^b^*wbeT* gene sequence was obtained from reference [16].

We hypothesized that *V*. *cholerae* O1 Inaba serotypes collected in 2013 (n = 2) and between October and December, 2015 (n = 30) (Table 2) from multiple sites (Fig 1) sustained distinct mutation(s) in *wbeT*. To test our hypothesis, we compared the *wbeT* gene sequences of these strains (Table 2) to Ogawa serotype of Haitian strains and prototype NIH41 strain [Table 2). Data indicate that an Inaba isolate (2013HC-380 (S1 Table)] collected in July, 2013 exhibited a substitution of A for G at nucleotide position 395 in *wbeT* gene resulting in an amino acid change from glutamate to glycine (Table 2); interestingly, another Inaba isolate [2013HC-795 (S1 Table)] collected in November 2013 sustained a deletion mutation (loss of C) at position 474 in *wbeT* gene resulting in the generation of a premature stop codon at position 481 resulted in a truncated WbeT protein (Table 2). Intriguingly, sequence analysis of *wbeT* gene of a representative group of Inaba isolates [n = 30 (S1 Table)] collected from clinical and environmental samples between October and December, 2015 revealed that a nucleotide C has been deleted from position 329 in *wbeT* gene resulting in the generation of a stop codon at 385 position that in turn created a truncated WbeT protein (Table 2).

In summary, our results indicate that, *wbeT* gene has received distinct mutations (point and deletion mutation) as of 2013 and these mutations have driven the serotype conversion of circulating Ogawa serotype to Inaba serotype with the recent acute spike in conversion. In regard to mutations in *wbeT* gene, our results are in contrast to a previous report [16] in which 5 Inaba isolates sustained a point mutation at position 493 (G→T) resulting in an immature stop codon at position 493 leading to a truncated WbeT protein (Table 2). Whereas compared to an earlier report [16], we observed distinct mutations in *wbeT* gene in our Inaba isolates, we reason that cause of such mutations in *wbeT* gene may be driven by similar selective pressure(s). Although, the underlying cause of the large number of mutations in *wbeT* gene within a short period of time has yet to be elucidated, it is tempting to speculate that human and/or environmental stressors have contributed to that serotype conversion. This evolving scenario highlights the need for ongoing clinical and environmental surveillance in Haiti, as we seek to understand drivers for the apparent resurgence of this epidemic. Only with such data we will be in a position to eradicate cholera in Hispaniola, as has been proposed by WHO, PAHO, and the Haitian Ministry of Public Health and Population.

## Supporting Information

S1 Table A list of *V*. *cholerae* O1 strains used in this study with their sources of isolation, dates and places of isolation in Haiti. HC, Haiti clinical; C, clinical (stool) samples; E, environmental (water) samples.
(DOCX)
