# Supplementary material for: Major Shift of Toxigenic V. cholerae O1 from Ogawa to Inaba Serotype Isolated from Clinical and Environmental Samples in Haiti
Source: PLoS Negl Trop Dis. 2016 Oct 7;10(10):e0005045. doi: 10.1371/journal.pntd.0005045 (PMC5055329; doi:10.1371/journal.pntd.0005045)
Supplement: S1 Table — HC, Haiti clinical; C, clinical (stool) samples; E, environmental (water) samples. (DOCX) [file pntd.0005045.s001.docx]

**S1 Table. A list of *V. cholerae* O1 strains used in this study with their sources of isolation, dates and places of isolation in Haiti.**

| Strain | Accession Number (references^a^) | Isolation date | Site of isolation | Sample source | Serotype |
| --- | --- | --- | --- | --- | --- |
| 2010AA-142 | JSDO00000000 (1) | November 9, 2010 | St. Marc Hospital, Artibonite, Haiti | C | Ogawa |
| 2010AA-151 | JSTB00000000 (1) | November 9, 2010 | St. Marc Hospital, Artibonite, Haiti | C | Ogawa |
| 2012HC-31 | JSTZ00000000 (1) | June 20, 2012 | Cholera Treatment Center, Gressier, Haiti | C | Ogawa |
| 2012HC-34 | JSUC00000000 (1) | June 20, 2012 | Cholera Treatment Center, Gressier, Haiti | C | Ogawa |
| 2012Env-90 | JSTI00000000 (1) | May 24, 2012 | Gressier, Haiti | E | Ogawa |
| 2012 Env-131 | JSTB00000000 (1) | June 22, 2012 | Gressier Beach, Haiti | E | Ogawa |
| 2013HC-494 | KX379842 | August 22, 2013 | Notredame Hospital, Petit Goave, Haiti | C | Ogawa |
| 2013HC-991 | KX379843 | December 15, 2013 | Cholera Treatment Center, Jacmel, Haiti | C | Ogawa |
| 2013Env-894 | KX379844 | June 27, 2013 | Brach,Leogane, Haiti | E | Ogawa |
| 2013Env-1231 | KX379845 | October 28, 2013 | Reserved, Gressier, Haiti | E | Ogawa |
| 2014HC-1582 | KX379846 | June 24, 2014 | Notredame Hospital, Petit Goave, Haiti | C | Ogawa |
| 2014HC-1702 | KX379847 | October 23, 2014 | Cholera Treatment Center, Gressier, Haiti | C | Ogawa |
| 2014Env-1792 | KX379848 | May 26, 2014 | Bay Larion bridge, Haiti | E | Ogawa |
| 2014Env-2067 | KX379849 | June 8, 2014 | Bay Larion bridge, Haiti | E | Ogawa |
| 2015HC-2039 | KX379850 | November 20, 2015 | Cholera Treatment Center, Jacmel, Haiti | C | Ogawa |
| 2015HC-2042 | KX379851 | November 20, 2015 | Cholera Treatment Center,Jacmel, Haiti | C | Ogawa |
| 2013HC-380 | KX379810 | July 16, 2013 | Notredame Hospital, Petit Goave, Haiti | C | Inaba |
| 2013HC-795 | KX379811 | November 6, 2013 | Cholera Treatment Center, Jacmel, Haiti | C | Inaba |
| 2015Env-4450 | KX379812 | November 24, 2015 | Colin, Gressier, Haiti | E | Inaba |
| 2015Env-4452 | KX379813 | November 24, 2015 | Sousfort, Gressier, Haiti | E | Inaba |
| 2015HC-1961 | KX379814 | October 15, 2015 | Cholera Treatment Center, Gressier, Haiti | C | Inaba |
| 2015HC-1975 | KX379815 | October 29, 2015 | Cholera Treatment Center,Jacmel, Haiti | C | Inaba |
| 2015HC-1978 | KX379816 | October 29, 2015 | Cholera Treatment Center, Jacmel, Haiti | C | Inaba |
| 2015HC-1979 | KX379817 | October 30, 2015 | Cholera Treatment Center, Gressier, Haiti | C | Inaba |
| 2015HC-1996 | KX379818 | November 5, 2015 | Cholera Treatment Center, Gressier, Haiti | C | Inaba |
| 2015HC-1998 | KX379819 | November 5, 2015 | Cholera Treatment Center, Gressier, Haiti | C | Inaba |
| 2015HC-2001 | KX379820 | November 11, 2015 | Cholera Treatment Center, Gressier, Haiti | C | Inaba |
| 2015HC-2003 | KX379821 | November 11, 2015 | Cholera Treatment Center, Gressier, Haiti | C | Inaba |
| 2015HC-2015 | KX379822 | November 12, 2015 | Cholera Treatment Center, Jacmel, Haiti | C | Inaba |
| 2015HC-2017 | KX379823 | November 12, 2015 | Cholera Treatment Center, Jacmel, Haiti | C | Inaba |
| 2015HC-2018 | KX379824 | November 12, 2015 | Cholera Treatment Center, Jacmel, Haiti | C | Inaba |
| 2015HC-2021 | KX379825 | November 13, 2015 | Cholera Treatment Center, Gressier, Haiti | C | Inaba |
| 2015HC-2030 | KX379826 | November 19, 2015 | Cholera Treatment Center, Gressier, Haiti | C | Inaba |
| 2015HC-2031 | KX379827 | November 19, 2015 | Cholera Treatment Center, Gressier, Haiti | C | Inaba |
| 2015HC-2033 | KX379828 | November 19, 2015 | Cholera Treatment Center, Gressier, Haiti | C | Inaba |
| 2015HC-2035 | KX379829 | November 20, 2015 | Cholera Treatment Center, Jacmel, Haiti | C | Inaba |
| 2015HC-2036 | KX379830 | November 20, 2015 | Cholera Treatment Center, Jacmel, Haiti | C | Inaba |
| 2015HC-2040 | KX379831 | November 20, 2015 | Cholera Treatment Center, Jacmel, Haiti | C | Inaba |
| 2015HC-2041 | KX379832 | November 20, 2015 | Cholera Treatment Center, Jacmel, Haiti | C | Inaba |
| 2015HC-2043 | KX379833 | November 20, 2015 | Cholera Treatment Center, Jacmel, Haiti | C | Inaba |
| 2015HC-2051 | KX379834 | November 25, 2015 | Cholera Treatment Center, Jacmel, Haiti | C | Inaba |
| 2015HC-2052 | KX379835 | November 25, 2015 | Cholera Treatment Center, Jacmel, Haiti | C | Inaba |
| 2015HC-2056 | KX379836 | November 26, 2015 | Cholera Treatment Center, Gressier, Haiti | C | Inaba |
| 2015HC-2057 | KX379837 | November 26, 2015 | Cholera Treatment Center, Gressier, Haiti | C | Inaba |
| 2015HC-2062 | KX379838 | November 26, 2015 | Cholera Treatment Center, Gressier, Haiti | C | Inaba |
| 2015HC-2068 | KX379839 | December 3, 2015 | Cholera Treatment Center, Jacmel, Haiti | C | Inaba |
| 2015HC-2074 | KX379840 | December 3, 2015 | Cholera Treatment Center, Jacmel, Haiti | C | Inaba |
| 2015HC-2081 | KX379841 | December 3, 2015 | Cholera Treatment Center, Gressier, Haiti | C | Inaba |

HC, Haiti clinical; C, clinical (stool) samples; E, environmental (water) samples.

^a^wbeT gene sequences was obtained from the indicated reference (see reference 1 below)

1. Azarian T, Ali A, Johnson JA, Mohr D, Prosperi M, Veras NM, et al. Phylodynamic analysis of clinical and environmental *Vibrio cholerae* isolates from Haiti reveals diversification driven by positive selection. MBio. 2014;5(6).
